# Dietary fat induces sustained reward response in the human brain without primary taste cortex discrimination

**DOI:** 10.3389/fnhum.2013.00036

**Published:** 2013-02-20

**Authors:** Hélène Tzieropoulos, Andreas Rytz, Julie Hudry, Johannes le Coutre

**Affiliations:** ^1^Perception Physiology Group, Food Consumer Interaction Department, Nestlé Research CenterLausanne, Switzerland; ^2^Applied Mathematics Group, BioAnalytical Science Department, Nestlé Research CenterLausanne, Switzerland; ^3^Organization for Interdisciplinary Research Projects, The University of TokyoTokyo, Japan

**Keywords:** calorie detection, electro-encephalography, gustatory event-related potentials, milk, salt

## Abstract

To disentangle taste from reward responses in the human gustatory cortex, we combined high density electro-encephalography with a gustometer delivering tastant puffs to the tip of the tongue. Stimuli were pure tastants (salt solutions at two concentrations), caloric emulsions (two milk preparations identical in composition except for fat content) and a mixture of high fat milk with the lowest salt concentration. Early event-related potentials (ERPs) showed a dose-response effect for increased taste intensity, with higher amplitude and shorter latency for high compared to low salt concentration, but not for increased fat content. However, the amplitude and distribution of late potentials were modulated by fat content independently of reported intensity and discrimination. Neural source estimation revealed a sustained activation of reward areas to the two high-fat stimuli. The results suggest calorie detection through specific sensors on the tongue independent of perceived taste. Finally, amplitude variation of the first peak in the event-related potential to the different stimuli correlated with papilla density, suggesting a higher discrimination power for subjects with more fungiform papillae.

## Introduction

Why is fatty food so appealing? Just as carbohydrates, minerals and proteins, fatty acids are essential to proper functioning of the body and survival. However, contrary to classic tastants for which involvement of the gustatory system has been demonstrated, the detection mechanisms for fatty acids and their central representation are still unclear.

Traditionally, fat detection has been considered to occur through texture, which makes food with high fat content unique in terms of mouthfeel (thickness, mouth coating, etc.). Texture, however, is not the only cue: olfaction contributes to the detection of fatty acids in animals, although evidence in humans is still controversial (Mattes, 2009). More recently, the role of the gustatory system and the hypothesis of fat taste have undergone deep investigation. Animal research has shown preferences for fatty foods (Laugerette et al., 2007) that were lost after *Chorda tympani* nerve transection (Stratford et al., 2006) but preserved in anosmic rats (Fukuwatari et al., 2003). At the molecular level, a diverse group of primary signal transduction molecules (i.e., fat taste receptors) has been described in the membranes of lingual tissue cells (Mattes, 2010). Although some of these mechanisms have been linked to human perception of fatty acids, preference or obesity (Galindo et al., 2012; Ichimura et al., 2012; Pepino et al., 2012), no conclusive evidence has been obtained for a fatty taste signal transduction mechanism in humans. Finally, neuroimaging studies have reported activations in taste and reward areas as a response to food with varying fat content (De Araujo and Rolls, 2004; Grabenhorst et al., 2010; Eldeghaidy et al., 2011). Two major issues have hampered clear conclusions about animal and human fat detection studies, both originating from the difficulty to separate somatosensory responses from taste responses. On one hand, thickening agents are often used to provide control conditions with the same viscosity as fat. Not only are these products rarely completely tasteless but viscosity is not the only characteristic of fat texture (creaminess, oiliness, slickness). Moreover, texture by itself can activate taste and reward areas (Rolls, 2011). On the other hand, free fatty acids as used in sensory research quickly oxidize and induce irritation, which in turn is again detected by the trigeminal system (Bryant and Moore, 1995). Indeed, fatty acids generally occur esterified with glycerol as triglycerides in dietary fat, and their free form is more often found in spoiled food. Therefore, the role of the induced sensations in humans could rather be to prevent ingestion of dangerous food than to support their selection (Drewnowski and Almiron-Roig, 2010; Mattes, 2011).

In humans, the primary gustatory cortex has been located in the insula and the rolandic and frontal operculi (Veldhuizen et al., 2011) where responses are modulated by stimulus properties such as quality and intensity (Spetter et al., 2010) but independent of the context (De Araujo et al., 2003). The secondary gustatory cortex has been located mainly in the orbitofrontal cortex (O'Doherty et al., 2001), a structure involved in the assignment of reward value to the stimulus and its relevance at a given moment (Rolls et al., 1989). This pattern is often observed for sensory event-related potentials (ERPs) in electroencephalography (EEG): early potentials largely reflect changes in physical properties of the stimulus whereas late potentials can be altered by the context and are linked to a second step of information processing such as value assessment or other cognitive processes (Coles and Rugg, 1996). So far, only a few EEG studies have addressed gustatory responses mainly because of the need for millisecond-precision in the stimulation (Ohla et al., 2012).

This dissociation in roles of primary and secondary gustatory cortices has been underlined in a study where differences in calorie content between two iso-sweet solutions were reflected in secondary but not in primary taste areas (Chambers et al., 2009). Moreover, a tasteless compound with the same calorie content as sugar triggered similar responses in reward areas but not in primary gustatory areas. This finding supported the assumption that apart from smell, texture, and taste, calorie detection might explain how energy-dense foods are recognized, selected, and ingested.

The main objective of the current study is to elucidate the relative contributions of taste and reward systems in the detection of fat from the tip of the tongue. We used a tastant-delivery device, which allows for isolation of taste from other sensory cues to investigate cortical and subcortical responses to dietary fat and pure tastants in the millisecond time domain amenable to EEG. We hypothesized that (1) a difference in taste intensity will trigger differences in early potentials originating from primary gustatory areas (insula, rolandic operculum, frontal operculum), whereas (2) a difference in calorie content will be reflected in late potentials originating from the secondary gustatory cortex (orbitofrontal cortex, anterior cingulate cortex). Finally, we hypothesized that (3) a difference in both taste intensity and calorie content will generate modulations of both early and late potentials.

## Materials and methods

### Tastant and milk preparations

Overall, two aqueous salt solutions were prepared differing in their concentration (hypothesis 1: High Salt vs. Low Salt). In addition two milk preparations were matched for their composition except for a 5% difference in fat content (hypothesis 2: High Fat vs. Low Fat). Finally, one mixture of the high fat milk preparation with the low salt concentration was prepared to be compared to the low fat milk (hypothesis 3: Low Fat vs. FatSalt).

Salt concentrations were chosen based on informal tasting sessions, with the constraints that stimuli had to be detectable by all participants yet also distinct, in a range where they do not trigger disgust or irritation. Fat concentrations were chosen to maximize fat difference between the two milk preparations while having the same viscosity.

Table salt was dissolved in 100 ml of low-mineralized water Acqua Panna (Sanpellegrino S.p.A, Milano, Italy) at two different concentrations [“Low Salt”: 0.8 g (130 mM) and “High Salt”: 4 g (690 mM)]. To obtain milks varying only in fat content, high fat and low fat milk powders (Emmi Group, Lucerne, Switzerland) were diluted into low-mineralized water after being matched for their carbohydrate contents. With this procedure, carbohydrate (lactose) content was 9% (w/v), protein content 4.5% (w/v), and mineral salt content 0.1% (w/v). Only the fat content varied from 0.03% (w/v) for the low fat milk to 5% (w/v) for the high fat milk. The mixture “FatSalt” was obtained by adding the “low salt” (130 mM) concentration to the “high fat” (5%, w/v) milk. To test viscosity, stimuli were heated at 37°C and measured with a rheometer Physica MCR 501, TEZ measuring cell, DG26.7 geometry. Pressure loss for each stimulus was calculated as follows:

$$\Delta p=\frac{1}{2}\;\cdot \;\rho \;\cdot \;u_{m}^{2}\;\cdot \;\frac{L}{D}\;\cdot \;\lambda_{\text{lam}},\;\lambda_{\text{lam}}=\frac{64}{Re_{D}}$$

were ρ is the density of the liquid, υ its mean velocity [volumic flow rate/(pi × radius^2^)], L the length of the device tube, and D its diameter. In this equation, λ is a coefficient of pressure drop which is equal to 64/Re for laminar flows. Volumic flow rate is mass flow rate/density and Re the Reynolds number. The viscosity difference between high fat and low fat milks was of 1 mPa·S at a shear rate of 100/s. This value resulted in a negligible pressure loss, confirming that all liquids in this setup behaved like water (0.0006 mPa for High Fat and 0.0003 mPa for Low Fat compared to 0.0002 mPa for water).

### Participants

Twenty healthy volunteers (10 men, 20–45 years old, average = 30.85) with a BMI in the normal range (average = 22.61), were recruited. All subjects gave their written informed consent, declared no history of taste/smell/neurological disorders and limited cigarette consumption (max 3/day). They were asked to refrain from alcohol consumption during the 24 h prior to each session. The day of the session, subjects had their usual breakfast and did not eat/drink anything but water until coming to the laboratory at 10 a.m. They were remunerated for their participation. This study was conducted according to the declaration of Helsinki and was approved by the local ethics committee (“Commission cantonale vaudoise d'éthique de la recherche sur l'être humain”).

### Gustometry-EEG

**Figure 1:**
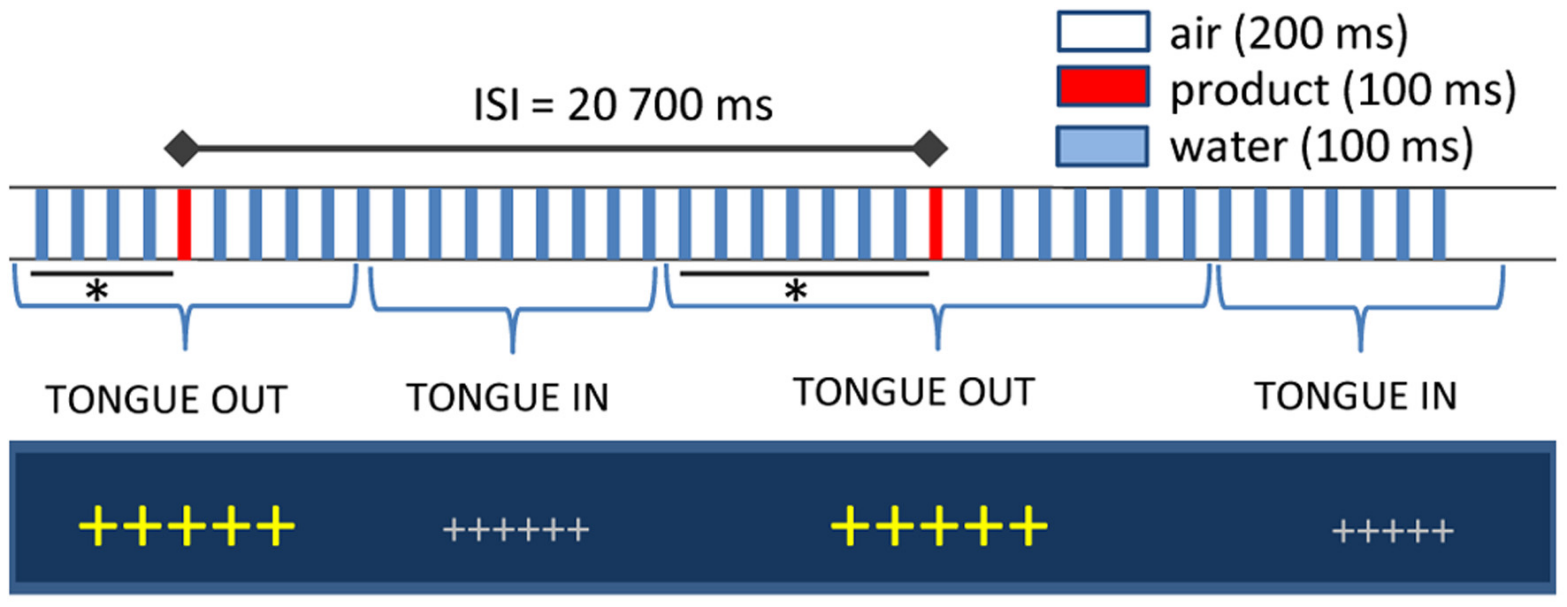
**Experimental setup.** Large fixation crosses indicated when participants had to position their tongue under spray head: the interval between cross size change and the actual delivery of the stimulus varied between 5 and 8 s (indicated with an asterisk). Inter-stimulus interval was fixed at 20.7 s. In between, water puffs of 100 ms were delivered every 300 ms and inserted in a stream of compressed air.

Liquid stimuli were delivered on the participants' tongues through a gustometer GU002 (Burghart, Wedel, Germany). A technical description of the device and of the stimulus properties is available upon request to the manufacturer (www.burghart-mt.de). Seventy μl of low-mineralized bottled water (Aqua Panna, San Pellegrino S.p.A, Milano, Italy) were continuously sprayed by a nozzle placed above the tongue of the participants every 300 ms for a duration of 100 ms (Figure 1). With an inter-stimulus interval of 20.7 s, one water puff was replaced by the taste stimulus. The computer-controlled gustometer was used to send the liquids, which were embedded into a constant flow of compressed air. Due to the high frequency of stimulation (3–4 pulses/s), water flow is perceived as continuous and therefore tactile responses in the brain are avoided. Temperature was fixed to 37°C and texture effects were abolished by the homogenous dispersion of the spray and immediate rinse off by subsequent water puffs. Fixation crosses on the computer screen changed color and size when participants had to place their tongue under the spray. The delay between tongue positioning and actual delivery of the stimulus varied between 5 and 8 s to avoid learning effects leading to expectations, to ensure somatosensory desensitization by water sprays before stimulus delivery and to allow for subsequent proper rinsing of the tongue. To control for other confounding sensory cues, nostrils were blocked by cotton buds and white noise delivered through headphones.

Due to the length and flexibility of the gustometer's tubes (5 m here), the effective liquid output has a certain delay time in relation to the electrical trigger signal. The tube length may also limit the stimulus rise time. The delay time was measured by the manufacturer as following (extract from the technical document provided by Burghart, Wedel, Germany): “The delay time is measured from the beginning of the electrical trigger signal to the point where the stimulus concentration has reached 5% of its maximum (relative scale). The rise time is defined as the time from 5 to 90% normalized concentration. One percentage NaCl solution is used to measure stimulus properties, because of its significantly different electrical resistance compared to water. The solution is applied with the gustometer on stainless steel electrodes which are facing each other with a gap. The change in electrical resistance between the background water and the taste solution is recorded with an oscilloscope to measure delay and rise time. Measuring electrical resistance of a salt solution with DC voltage leads to depletion of ions (which are the charge carriers and thus determine the electrical resistance of the solution) close to the electrodes, which results in increasing resistance and thus an increasing measurement error. AC voltage prevents ion depletion. For that reason, a frequency generator with 10 kHz is used as voltage source and the envelope of the AC recording is the stimulus signal. The measurement resistor is 10 kΩ. The peaks of the AC recording (the envelope) show the change in conductivity over time. All measurements are offset-corrected and normalized (peak of envelope is set to 1.0, i.e., 100%). Measurements show that results for delay time depend on the following parameters:

– Pulse flow rate *Q*_*P*_, calculated by dividing the pulse volume (*Pv*) by the pulse duration (*Pd*). The pause duration is unimportant for the delay time: *Q*_*P*_ = *Pv*/*Pd*;
– connection hose length (5 m or 12 m);
– use of spray head or “open” use.”

With this method, the delay time was estimated to 36 ms all parameters considered. Therefore, all measures related to time were corrected for this delay as described in the “Procedures” section.

A BioSemi Active-Two amplifier system was used to conduct the EEG recordings (BioSemi, Amsterdam, The Netherlands). A 64 Ag/AgCl electrodes set was fixed to a cap provided by the manufacturer and mounted according to the extended 10–20 system. A common mode sense (CMS) active electrode is used as a reference and a passive DRL (driven right leg) electrode as a ground. Data were recorded at a sampling frequency of 512 Hz (fifth order sync filter with a −3 dB point at 1/5th of the sampling frequency) and stored on hard disk for later off-line analysis.

### Procedures

Participants were told that different components of milk will be tested but they were not aware of the exact composition of each stimulus. For the three sessions described below, participants were installed in a sound-attenuated, shielded recording booth, and seated at approximately 80 cm of a computer screen.

The goal of session 1 was to familiarize participants with the gustometer as well as to collect data on the stimuli and on the participants' papilla density. In a first block of 10 stimulations (each stimulus in duplicate), participants were instructed how to be positioned to optimize stimulus perception. This first block also allowed ensuring that all stimuli were detected thanks to an immediate feedback to the experimenter. In the two following blocks of 25 stimulations, reaction times (RTs) for detection were measured (50 stimulations in total, i.e., 10 *per* stimulus in a pseudo-random order). In a final block of 10 stimulations, ratings of pleasantness and intensity were done on a seven-point scale (2 repetitions/stimulus). Subjects were also asked to indicate which of the five basic tastes the stimulus resembled the most (salty, sweet, bitter, sour, umami). This forced-choice procedure was adapted from classical procedures used to assess gustatory function (e.g., Landis et al., 2009); it was chosen for its rapidity and simplicity of execution by naive participants. The aim was to get a clear insight on the dominant taste quality of the stimuli perceived by the participants without extensive sensory profiling. To determine papilla density, a blue food colorant was applied with a cotton bud on the tip of the tongue, an area corresponding to where the stimuli reached the tongue during the experiment. Participants were then seated with their heads positioned on a chin-rest. A plastic frame of 2.5 cm^2^ was deposited on the colored area and a picture was taken with a digital camera (Leica D-Lux4, macro mode with 9 mio pixel resolution) fixed on a tripod. The blue colorant did not stain the fungiform papillae, which remained red (Miller and Reedy, 1990). Counting was done twice and since the coefficient of variation was very low (4%), mean counts were further analyzed.

Sessions 2 and 3 were identical, each composed of 5 blocks of 30 stimulations (150 stimulations/session), for a total of 300 stimulations (60 *per* taste stimulus). Presentation order was pseudo-randomized, such that over the five blocks each stimulus was preceded six times by the 4 other stimuli and 5 times repeated. At the end of session 3, intensity, pleasantness ratings, and dominant taste quality were assessed again (2 repetitions/stimulus). The exact composition of the five taste stimuli was then revealed to the participants and their ability to discriminate among them was tested using a five-alternative-forced-choice recognition task (each stimulus in duplicate).

### Electrophysiological data analysis

Epochs of 936 ms (100 ms pre and 836 ms post stimulus) were extracted and computed for each stimulus category and each participant after DC removal, notch filtering at 50 Hz and superior harmonics until 250 Hz (Nyquist frequency) and high-pass filtering at 0.1 Hz. Trials with eye blinks were rejected based on visual inspection of each epoch individually. Four subjects (three men) were withdrawn from the data set because neither the first ERP component (i.e., P1) to High Salt nor the corresponding scalp topography could be detected (mainly because of high contamination by alpha waves). Average epochs aligned on the onset of stimulation were computed for all 16 remaining subjects and stimuli. Stimulus onset was corrected for 36 ms because of an instrument specific delay between the trigger and actual delivery of the stimulus, resulting in a pre-stimulus period of 136 ms and post stimulus period of 800 ms.

**Figure 2:**
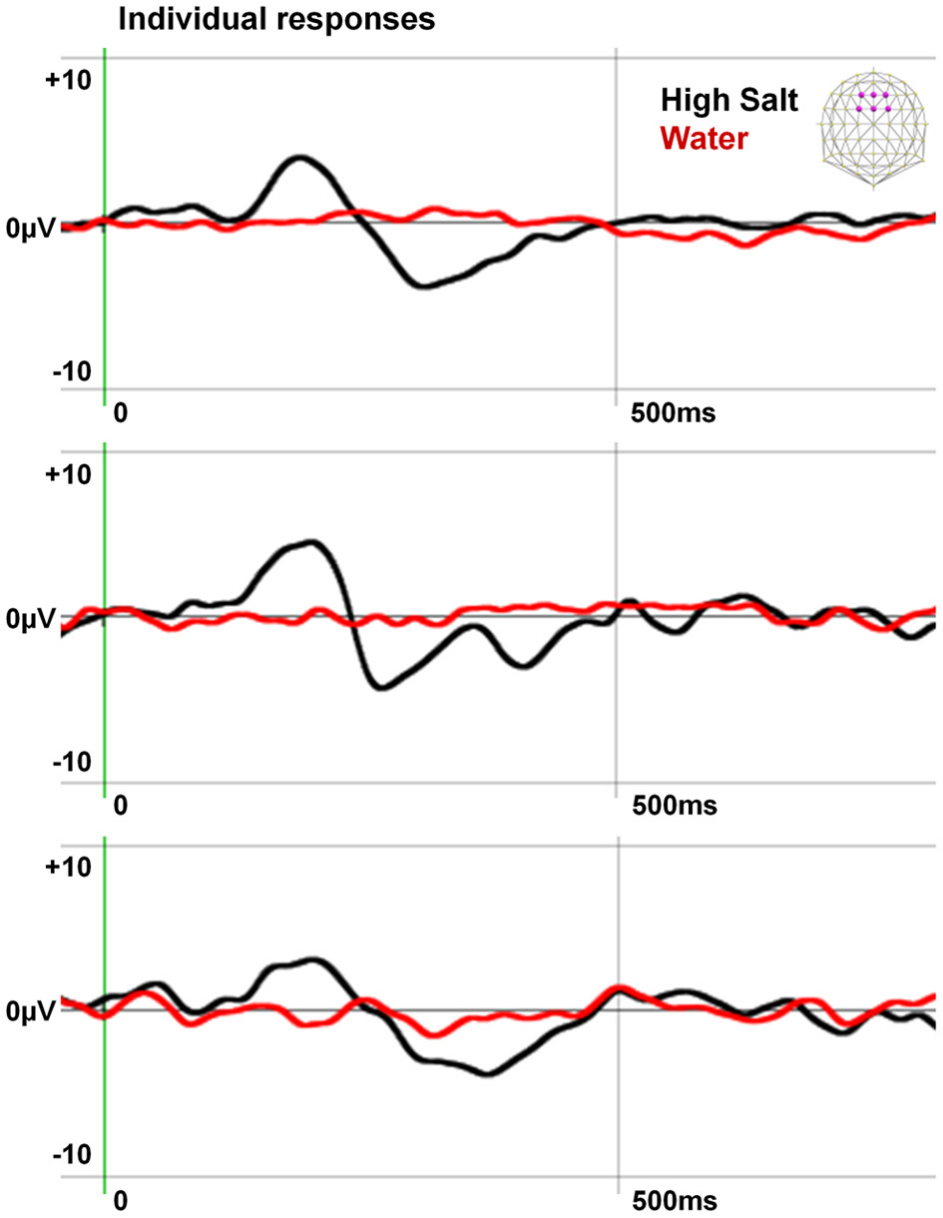
**Specificity of the EEG responses induced by the gustometer.** The individual responses of three subjects to water puffs and High Salt stimuli are averaged on six fronto-central electrodes indicated in purple on the schematic scalp top right. Data, filtered at 20 Hz for the display, show an ERP to the High Salt stimulus but not to the water spray demonstrating the gustatory nature of this response.

To confirm the specificity of the gustometer to produce pure gustatory responses without trigeminal contamination, we extracted and averaged epochs aligned on the water puffs preceding High Salt stimulations by 1200 ms when the tongue of the participant was in place (see Figure 1). The individual responses obtained in three participants are presented with those computed for the High Salt stimulus in Figure 2.

Stimuli were tested according to the three hypotheses: Low vs. High Salt, Low vs. High Fat, and Low Fat vs. FatSalt following standard procedures (Brunet et al., 2011) implemented in the Cartool software by Denis Brunet (brainmapping.unige.ch/cartool). All statistics were done on average-referenced data.

**Figure 3:**
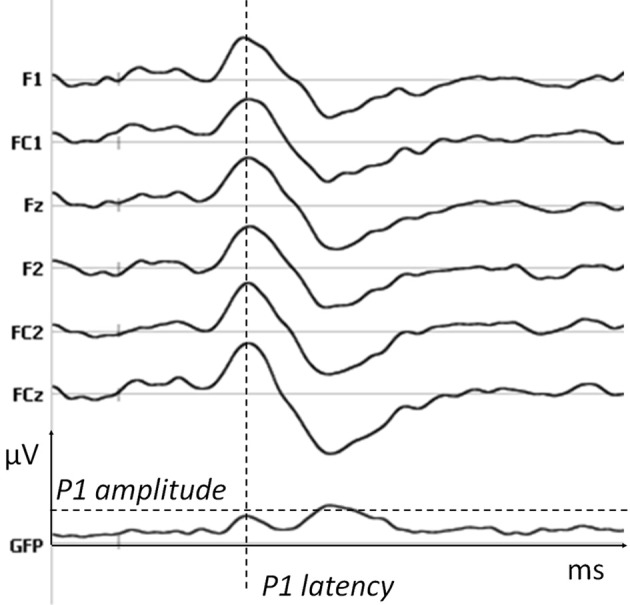
**P1 parameters.** The amplitude and latency of P1 for each subject were extracted from the maximal value of GFP in the time window of the first positive peak. An example is shown here on one subject and six fronto-central electrodes on which P1 is maximal (data filtered at 20 Hz for the display).

First, amplitudes were compared electrode by electrode and time point by time point using parametric *t*-testing. To correct for multiple testing a time constraint was added (differences longer than 20 ms consecutively, i.e., 10,240 data points) and a significance level of 0.01 was applied. *T*-tests with identical constraints were applied on Global Field Power (GFP), allowing the detection of differences in global energy between conditions. GFP is a measure of electric field power at each time point, which is calculated as the root mean square across all electrodes at each time point and is therefore independent of the chosen reference. To ensure that differences in amplitudes were not due to a shift in latency, P1 amplitude (as defined by the corresponding maximal value of GFP, see Figure 3) was extracted and compared across conditions with *t*-tests. Map topographies were compared using the global dissimilarity as implemented in Cartool software. Importantly, differences in topographies necessarily imply at least one difference in the underlying generators, whereas the opposite is not true.

Second, microstate segmentation was used to define maps of stable topographies. This clustering method (Topographic Atomize and Agglomerate Hierarchical Cluster) identifies periods during which the topography remains identical and divides grand average data accordingly. Clusters correlating above 85% were merged and each template map had to last at least 30 ms. The choice of optimal segmentation was based on the Krzanowski-Lai criterion. This analysis allows a summarized representation of the group average data, therefore the presence of these maps at the subject level is tested in a second step (fitting procedure). The output of this procedure was set to give the timing of maximal GFP and the duration of each template map and every participant, allowing the use of paired *t*-tests to compare these parameters across conditions.

Finally we applied Local Auto-Regressive Average (LAURA) distributed linear inverse solution (Grave de Peralta Menendez et al., 2004) on periods of interest as defined by segmentation and topographical comparisons (TANOVAs) to estimate neural generators involved in every step of gustatory information processing. The solution space was calculated on a realistic head model (SMAC) with 3005 solution points. The points are equally distributed within MNI average brain (Montreal Neurological Institute). These estimations provide visualization of the generators, not statistical comparisons.

### Behavioral data analysis

Mixed effect models with stimuli as fixed and subjects as random effect were conducted on all behavioral data. Fisher's Least Significant Difference (LSD) on a 5% significance level was used for exploratory *post-hoc* analysis. This procedure was chosen to follow the *t*-test approach applied in the EEG data analysis. Prior log-transformation and 36 ms onset correction were applied on reaction times (Box-Cox transform). Only post-test scores of intensity and pleasantness were analyzed as they were taken once the subjects were familiar with the stimuli and the setup (no missing values due to a bad synchronization of the participant with the gustometer). For this same reason only post-test responses to “dominant taste” are described.

### Correlations

To investigate correlations between behavioral, anatomical, and electrophysiological measures two supplementary analyses were performed. A first analysis aimed to define the potential impact of four covariates (papilla density, age, BMI, and gender) on six outcomes of interest: RTs, P1 latency, amplitude of GFP at P1, intensity score, pleasantness score, and correct recognition. These six outcomes were summarized as follows for each subject: mean over the five stimuli as well as the range over the five stimuli (to assess variability of the responses). Pearson correlations between covariates and the 6 × 2 summarized outcomes were used to describe linear relationships. A second analysis aimed to define how the six outcomes of interest were related at the individual subject level. Individual Pearson correlations (on the five stimuli) were computed for the six outcomes. Median correlations (rMed) were then computed by taking the median on the 16 analyzed subjects.

## Results

### Taste stimulus concentration impacts both amplitude and latency of P1

**Figure 4:**
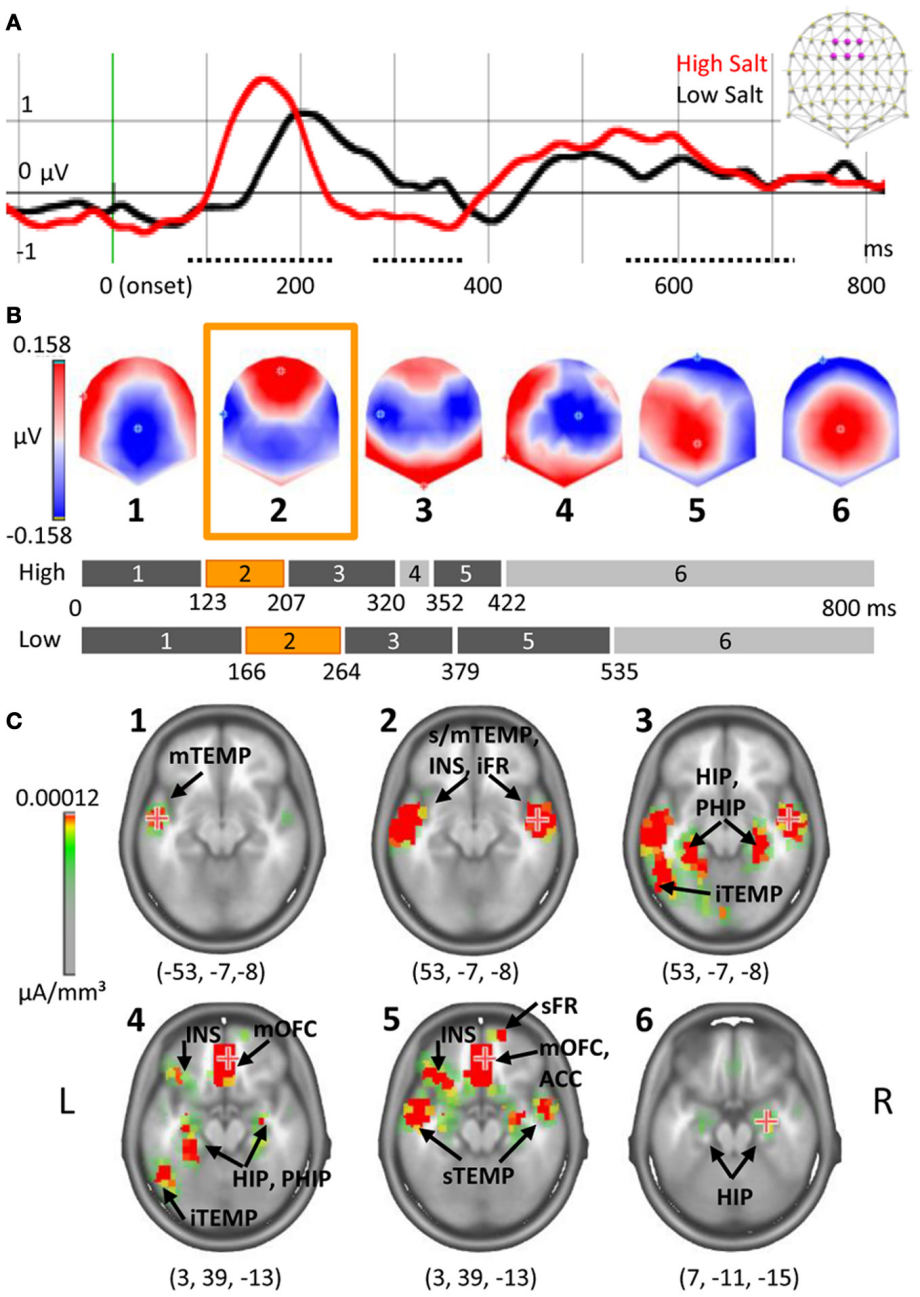
**Dose-response and chronometry of taste response to salt. (A)** ERP on the average of six fronto-central electrodes indicated in purple on the schematic scalp top right and filtered at 20 Hz for the display. Periods of amplitude differences are indicated with a dotted line, onset in green. **(B)** Segmentation based on six maps of stable topographies. The duration of each map is indicated on the bar graph, in orange the map corresponding to P1. **(C)** Estimation of neural generators for each of the template maps with coordinates of maximal activation. Note that scale was adjusted to see only the most active sources. The numbers in brackets indicate Talairach coordinates (*x*, *y*, *z*) of maximal activations. TEMP, temporal gyrus, INS, insula, FR, frontal gyrus, HIP, hippocampus, PHIP, parahippocampal gyrus, OFC, orbitofrontal cortex, ACC, anterior cingulate cortex; i/m/s, inferior/middle/superior.

To establish the cortical signature of a pure gustatory dose-response, salt solutions of different concentrations were tested. The amplitude of ERPs measured after High Salt and Low Salt stimulations revealed three periods of significant differences (*t*-test, >20 ms, *P* < 0.01) corresponding to each peak of the gustatory ERP (gERP) previously described (Mizoguchi et al., 2002; Ohla et al., 2010). For the first peak P1 differences were observed between 77 and 235 ms on 19 fronto-central electrodes, on eight fronto-central electrodes for the second peak N1 between 284 and 384 ms, and on 24 fronto-central and parietal electrodes for the last peak LPC (Late Positive Component) between 554 and 729 ms. Figure 4A shows the ERPs elicited by High Salt and Low Salt on the average of six fronto-central electrodes (F1, Fz, F2, FC1, FCz, FC2) where the amplitude of P1 was maximal. As electrodes were compared time point by time point, we verified that no differences in amplitude were created by the latency shift. For this, P1 amplitude and latency were determined by using the corresponding maximum GFP value (GFP i.e., the power of all electrodes taken together) for each subject individually as shown in Figure 3 for one subject. Subsequent *t*-testing on both values revealed significantly higher (*P* = 0.02) and faster (*P* < 0.01) response for High Salt compared to Low Salt condition. Differences in the GFP were also found during time windows of the other peaks N1 and LPC (340–450 ms, 650–712 ms, *P* < 0.01).

### Temporal dynamics of taste response

Microstate segmentation identified six periods of stable electric fields (template maps). One map (#4) was observed only in the High Salt condition (Figure 4B). The presence of each of these maps was then statistically compared to individual data for each condition, revealing that the number of time frames during which map 4 was observed was not significantly different across the two conditions.

The estimation of underlying generators based on each of these template maps revealed early activation of primary gustatory areas (Figure 4C) lasting in the three first template maps (until 320 ms for High Salt, 379 ms for Low Salt). For the first map, Talairach coordinates (*x*, *y*, *z*) indicated maximal activation in left middle temporal gyrus (−53, −7, −8) extending to the insula, frontal operculum, rolandic operculum, and superior temporal gyrus. The maximum shifted to the right superior temporal gyrus in the second map (53, −7, −8) with activation of adjacent areas and lingual gyrus. This maximal activation was maintained in map 3 with a more posterior network recruited including left superior, middle and inferior temporal gyri, but also hippocampus and parahippocampal regions (bilaterally). A shift toward anterior parts of the brain comprising the secondary gustatory cortex was observed starting with map 4 and lasting until the end of the time window. Map 4 occurred at the transition from residual activations of primary gustatory areas and parahippocampal gyrus observed in previous maps (#1–3) to new activations with a maximum in the right middle orbitofrontal cortex (OFC, 3, 39, −13). Main active areas comprised inferior frontal operculum on the left, triangular part of inferior frontal gyrus, and superior frontal gyrus on the right as well as inferior orbitofrontal and anterior cingulate cortices on the left. Map 5 preserved the same maximal value in right middle OFC (3, 39, −13) and encompassed the same network of pre-frontal regions but not posterior regions anymore. Finally the maximal activity shifted to the right hippocampus (7, −11, −15) for the last and longest map.

### Fat detection

**Figure 5:**
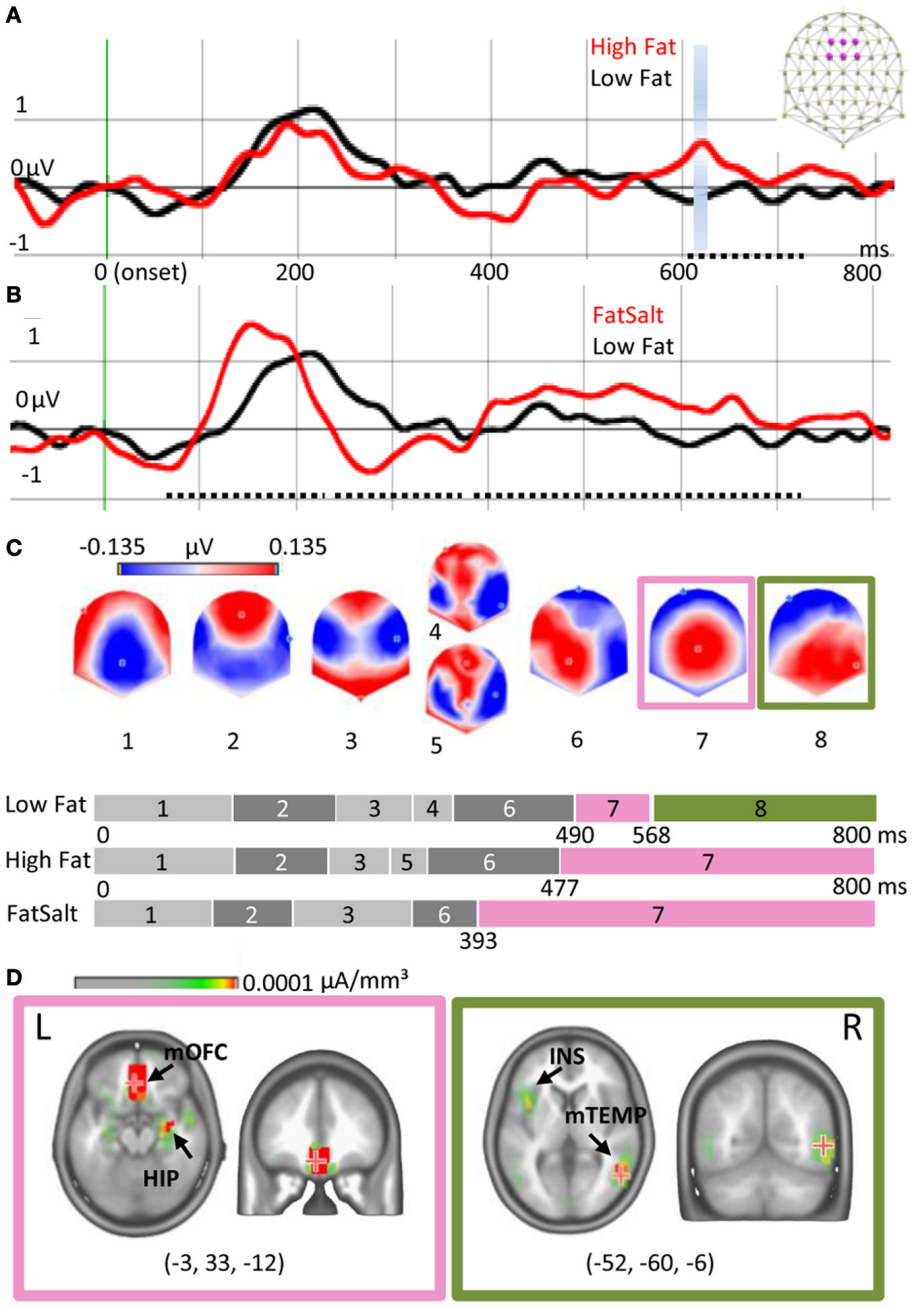
**Cortical and subcortical responses to milk emulsions. (A)** ERP for High- and Low-Fat and **(B)** for Low Fat and FatSalt, average of six electrodes indicated in purple on the schematic scalp and filtered at 20 Hz for the display, stimulus onset in green. Amplitude differences are indicated with dotted lines and topography differences highlighted blue. **(C)** Segmentation performed on the three conditions. Last part of the segmentation shows a different map for the Low Fat condition, as compared to the High Fat and FatSalt conditions, from 568 ms to the end of the time window consistent with topographical analysis. **(D)** Source estimation for maps 7 and 8 reveals sustained activation of reward-related areas for the stimuli containing fat but not for the low fat milk. The numbers in brackets indicate Talairach coordinates (*x*, *y*, *z*) of maximal activations. HIP, hippocampus; mOFC, middle orbitofrontal cortex; INS, insula; mTEMP, middle temporal gyrus.

Figure 5A shows the ERPs elicited by High Fat and Low Fat on the average of six fronto-central electrodes (F1, Fz, F2, FC1, FCz, FC2) where the amplitude of P1 was maximal. Amplitude comparison on all electrodes revealed no differences in the early ERP components, but a higher amplitude for High Fat compared to Low Fat between 603 and 714 ms post-stimulation (dotted line Figure 5A). This difference was significant (>20 ms, *P* < 0.01) on five neighbor fronto-central electrodes (F1, C1, FCz, Cz, C2). No differences were found for the GFP. As no shift of ERP latencies was observed, a TANOVA was performed, revealing a significant difference in topographies in the same interval but shorter in duration (612–622 ms, *P* < 0.01), highlighted blue in Figure 5A. During this interval, the last map for the High Fat condition was distributed around a positive maximum over central electrodes whereas the map for Low Fat showed a posterior positivity (see maps 7 and 8 of Figure 5C). This difference was confirmed by segmentation analysis on the two conditions offering a model of eight template maps. Template maps diverged from 537 ms to the end of the time window.

By contrast, amplitude comparison between Low Fat and FatSalt revealed amplitude differences in P1 between 65 and 237 ms (F1, C1, C5, T7, C2), N1 between 237 and 384 ms and large differences in LPC between 385 and 720 ms, respectively on 11 and 20 fronto-central and parietal electrodes (dotted lines Figure 5B). During the same time windows differences in GFP were also found (>20 ms, *P* < 0.01). As a shift in latency was again observed we compared P1 amplitude for each subject based on GFP maximal value, revealing a higher amplitude (*P* = 0.01) for FatSalt compared to Low Salt condition. Because of this latency shift, microstate segmentation was directly applied rather than a time-frame by time-frame TANOVA. Microstate segmentation on Low Fat vs. FatSalt yielded a model with seven template maps. As for the comparison between High- and Low-Fat, template maps diverged from 547 ms post stimulation until the end of the time window. The last map for FatSalt condition showed a large central positivity whereas the last map for Low Fat showed posterior positivity.

To verify the similarity between the last map of High Fat and the last map of FatSalt conditions we performed a general segmentation including the three conditions. The result (Figure 5C) confirmed that the last map (#7) for High Fat and FatSalt conditions was identical and dissimilar to the last map (#8) of Low Fat condition. In this model with 8 periods of stable topographies, one map (#8) was observed only in Low Fat condition. The presence of each of these template maps was then compared to subjects' individual data. Pairwise *t*-tests in the interval comprising maps 7 and 8 confirmed that on one hand, presence of map 8 was significantly higher in Low Fat compared to High Fat and FatSalt conditions, whereas on the other hand, map 7 was significantly more present in High Fat and in FatSalt compared to Low Fat condition (for all comparisons *P* < 0.01). The presence of the “transition” maps 4 and 5 did not differ between High and Low Fat but the difference reached significance level for the High Fat vs. FatSalt condition (*P* < 0.01).

Source estimation was done on maps 7 and 8 as they were consistently different across conditions and as this difference was confirmed by topographical analysis. Figure 5D shows the two different networks revealed by LAURA inverse solution with maximal value on left middle orbitofrontal cortex (−3, 33, −12) and hippocampus for map 7 and maximum on right middle temporal gyrus (52, −60, −6) and insula for map 8. Sources estimations were also calculated for maps 4 and 5 but they shared similar maximal value in the left superior temporal gyrus (−57, −32, −20) and the other sources were also found in maps 3 and 6, confirming their status of “transition maps.”

### Behavioral results

**Figure 6:**
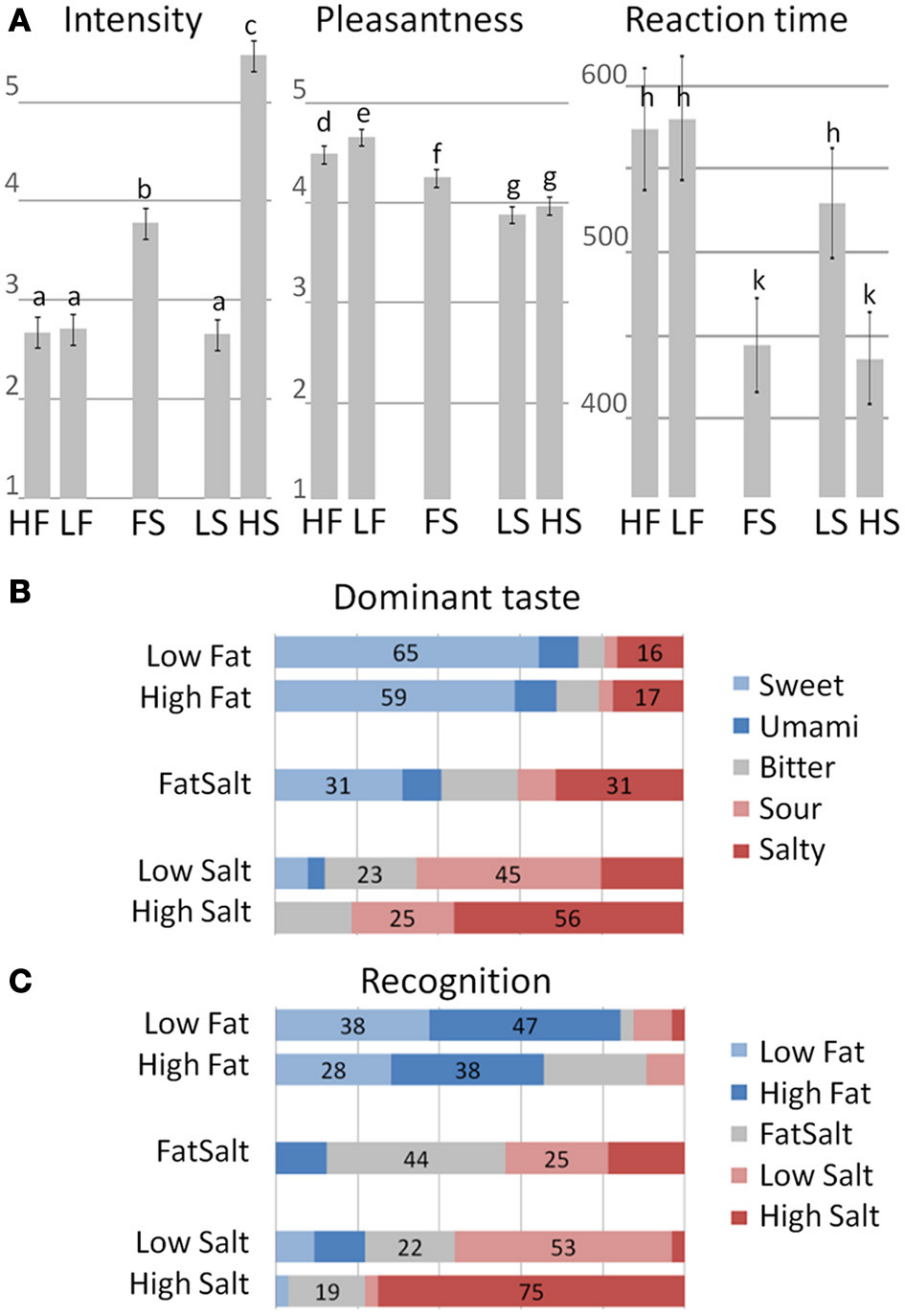
**Behavioral results. (A)** Intensity, Pleasantness ratings, and RT for the five stimuli. Error bars represent LSD and similar letters represent clusters with no differences. Intensity ratings: High Fat = 2.71, Low Fat = 2.6, FatSalt = 3.78, Low Salt = 2.66, High Salt = 5.47. Pleasantness ratings: High Fat = 4.66, Low Fat = 4.48, FatSalt = 4.27, Low Salt = 3.88, High Salt = 3.97, Reaction Times: High Fat = 580 ms, Low Fat = 573 ms, FatSalt = 444 ms, Low Salt = 529 ms, High Salt = 436 ms. **(B)** Distribution of the dominant taste descriptions with stimuli on the left and response given by the subjects on the right (forced-choice between five basic tastes). The numbers correspond to the percentage of the two most frequent answers. **(C)** Distribution of the recognition scores with identity of the stimuli on the left and responses given by the subjects on the right. The numbers correspond to the percentage of the two most frequent answers.

Intensity ratings clustered the five stimuli into three significantly distinctive groups (mixed effect model and *post-hoc* LSD on a 5% significance level), with higher mean score for High Salt compared to the four other stimuli as shown in Figure 6A, left panel. Pleasantness ratings also clustered the stimuli into three groups, with the highest pleasantness for the two milks followed by FatSalt and then the two equally pleasant salt solutions. The difference between High Fat and Low Fat milks was right at the significance level (Figure 6A, middle panel). Finally, reaction times were in line with intensity ratings with significantly shorter RTs for the two most intense stimuli than for the three others (Figure 6A, right panel).

Quality judgment on taste dominance (forced-choice between five basic tastes) was largely sweet for the two milks with no significant distinction between them (Figure 6B). FatSalt was described equally as salty or sweet by the participants, High Salt as predominantly salty and Low Salt as sour. Figure 6C shows the results of the forced-choice recognition task between the five stimuli, after participants were made aware of their identities. High Salt was the most easily identifiable stimulus (75% correct identifications), followed by Low Salt and FatSalt. Low Fat and High Fat milks were globally recognized as “milk” but not significantly distinguished from each other.

Correlation analyses revealed that, at the individual level, the lower the subject rated intensity of the stimulus, the higher the reaction time. This negative correlation between RTs and rated intensity was systematic for all subjects (*r*_Med_ = −0.72, *P* = 0.002). Similarly, a systematic negative correlation was observed between RTs and amplitude of P1 (*r*_Med_ = −0.68, *P* = 0.004). Finally, amplitude of P1 was systematically positively correlated with rated intensity (*r*_Med_ = 0.54, *P* = 0.031) and negatively correlated with latencies of P1 (*r*_Med_ = −0.59, *P* = 0.016).

**Figure 7:**
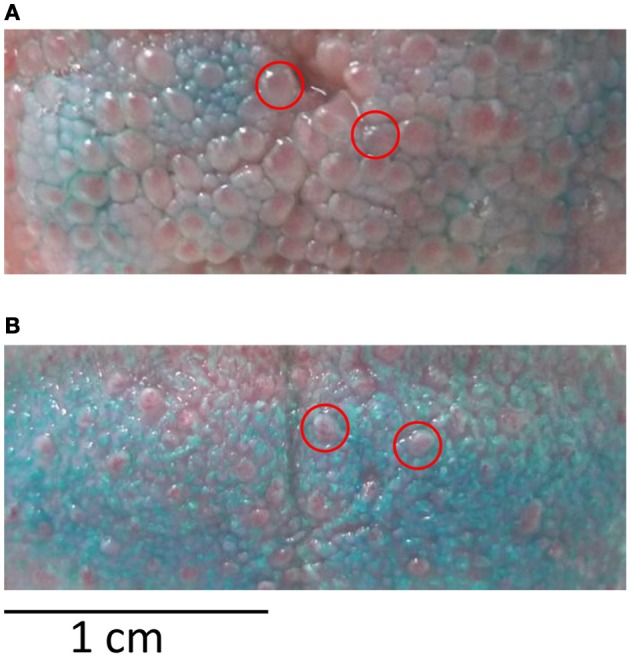
**Papilla density. (A)** Tongue with high papilla and **(B)** low papilla density after staining with blue food colorant. Red circles indicate exemplar locations of fungiform papilla that were counted in the colored area of 2.5 cm^2^.

Papilla density varied from 47 to 138 (average = 83) in a frame of 2.5 cm^2^ deposited on the tip of the tongue (Figure 7). All stimuli considered together, subjects with high papilla density scored the intensity lower on average (*r* = −0.50, *P* = 0.049), but presented greater variation in P1 amplitude to different stimuli (*r* = 0.57, *P* = 0.02). Male subjects had longer P1 latencies than female (*P* = 0.035). Increased age significantly correlated with lower mean intensity scores (*r* = −0.63, *P* = 0.009) and higher pleasantness scores (*r* = 0.51, *P* = 0.044). Increased BMI—although in the normal range—correlated with lower recognition scores (*r* = −0.51, *P* = 0.044) and with lower variation in pleasantness scores (*r* = −0.54, *P* = 0.031).

## Discussion

### Dose-response and signal latency

Salt concentration impacted both amplitude and latency of the P1 component of the gERP. The modulation of primary gustatory responses by concentration has already been reported in single neuron recordings with increased spiking rate as a function of concentration (Scott et al., 1991). A similar effect has been reported in human neuroimaging functional studies with changes in activation level (Small et al., 2003; Spetter et al., 2010), but EEG and magnetoencephalography (MEG) studies have yielded inconsistent results. With EEG, the effect of concentration on both the gERP's amplitude and latency has been reported for acetic and citric acids (Kobal, 1985; Hummel et al., 2010) but not for MSG and NaCl (Singh et al., 2011). Three MEG studies using NaCl reported effects of concentration on amplitudes and reaction times (Saito et al., 1998; Kobayakawa et al., 2008, 2012) but failed to observe an effect on latencies. Many factors can account for these inconsistencies such as the number of participants, of repetitions and of electrodes, as well as stimulus concentrations and presentation paradigm (same block or separately). Our results are consistent with a previous study using electrical taste (Ohla et al., 2010) where latency of the first peak was also impacted by stimulus intensity. Moreover, this effect has been reported in other sensory modalities: brightness on visual ERPs (Wicke et al., 1964); volume on auditory ERPs (Jaskowski et al., 1994); and concentration on olfactory ERPs (Pause et al., 1996; Tateyama et al., 1998). In our data set, concentrations were also impacting behavioral measures: at the group level, reaction times were shorter and intensity ratings higher for higher concentrations of salt. Results at the individual level further confirmed the relationships between RTs, intensity scores, and P1 parameters. Indeed, reaction times were negatively correlated with amplitude of P1 and reported intensity and positively correlated with latency (the shortest the RT, the shortest P1 latency, the highest the amplitude and the reported intensity).

Early potentials modulated by concentration originated mainly from middle and superior temporal gyri, insula and adjacent operculi (frontal and rolandic). This is consistent with previous reports of early primary gustatory cortex responses triggered by electric taste stimulation (Ohla et al., 2010). This early network also included other regions already described in taste research: hippocampus and parahippocampal gyrus (Tataranni et al., 1999); cuneus and lingual gyrus (Kinomura et al., 1994; Haase et al., 2011). Analysis of GFP revealed that not only early potentials but also late responses were impacted by concentration. Source localization confirmed the involvement of secondary gustatory cortex in this second processing step. Thus, the effect of concentration seems not restricted to the very first components of the gERPs but impacts the entire response. Nevertheless, the sources were identical for both salt solutions suggesting a similar information processing.

### Fat content

To disentangle the representation of taste from the representation of calories in the brain, the responses to the three milk conditions were analyzed together. The best model showed no early significant difference across conditions. However, a late template map, which was short-lived in the Low Fat condition lasted until the end of the time window for High Fat and FatSalt conditions. The most active sources for this map were located in the OFC and hippocampus. Fat content therefore affected responses in late potentials originating from reward/secondary gustatory areas but not early stages of the responses originating from primary gustatory areas.

Indeed contrary to salt solutions, ERPs to the high- and low-fat milks did not show a difference in the early potentials nor in GFP. This result was mirrored by behavioral data showing no differences in intensity ratings or in qualitative descriptions between the two milk preparations. Even when subjects were aware of the composition of the milk stimuli they were not able to discriminate amongst them. This collection of results in the context of our experimental setup (stimulation restricted to the tip of the tongue and 5% fat difference) excludes not only that fat acted as a taste but also and *de facto* that fat acted as a taste enhancer on the other components of milk.

For this same reason, the amplitude difference in early potentials in the comparison between Low Fat and FatSalt conditions (“adding taste and fat”) cannot be explained by an effect of taste enhancement due to higher fat content but is clearly the result of adding taste (salt) in the high fat milk. On the other hand, the difference in late components might as well be explained by the increased taste (as seen for salts) than by the effect of adding fat (as seen in milks), which is why the analysis of the three conditions together was crucial to confirm the responsibility of fat increase in these late differences.

Thus, late potentials alone were affected by fat content. To our knowledge, the modulation of late gustatory potentials by relevance or motivation has been reported once with an effect of sweet taste compared to neutral taste on P3 starting 400 ms post stimulation onset and related to self-reported food craving (Franken et al., 2011). Here, the late component reflected a second step of information processing originating from secondary gustatory cortex. As the sustained activation of OFC was observed not only for high-fat milk but also for FatSalt, despite its lower score on pleasantness scale, it is more probable that this step corresponds to stimulus value assessment (i.e., calorie content) rather than pleasantness assessment alone.

In a related study on the reward responses to sweet taste, saccharin and glucose solutions rated equally sweet and pleasant evoked similar responses in the right insula/frontal operculum and left dorsolateral prefrontal cortex (Chambers et al., 2009). Maltodextrin (without sweet taste but with similar calorie content as glucose) triggered responses in right insula/frontal operculum, medial orbitofrontal cortex, dorsolateral prefrontal cortex, right caudate and rostral ACC. This led to the hypothesis that calorie content rather than taste *per se* might be partly responsible for the responses in these areas. Importantly, the maltodextrin solution was not reported to be as pleasant as the glucose solution, similar to our study where FatSalt was reported less pleasant and High Fat more pleasant than the low-fat milk, supporting the hypothesis that the hedonic value of the stimulus cannot account for these differences. Another study (Frank et al., 2008) similarly showed that sucrose elicited higher responses in anterior insula, frontal operculum, striatum, and ACC than sucralose and also that only sucrose activated dopaminergic midbrain areas (VTA, substrantia nigra). Finally, a case study described a patient with severe destruction of the gustatory cortex (in particular insular cortex) who showed preference for sweet over salty solution without recognizing either and without being able to explain his choices (Adolphs et al., 2005).

Independent studies on fat perception argued in the direction of a fat-specific response reporting a positive correlation between fat level (from 5 to 30%) and brain activity in a network encompassing both primary and secondary gustatory areas (right anterior insula, bilateral frontal operculum right, ACC, amygdala) but also the somatosensory cortex (Eldeghaidy et al., 2011). The subjects showed preference for high fat samples and found them significantly different in terms of oiliness, therefore texture could partly account for these activations. Correlations between pleasantness of fat texture and activity in mid-orbitofrontal cortex and pregenual/anterior cingulate have been reported, along with differences between high and low fat flavored milks in hypothalamus, amygdala, and ventral striatum (Grabenhorst et al., 2010). However, the texture was not controlled and as it can activate neurons in the orbitofrontal cortex (Rolls, 2011) where information from different sensory cues converge to form a coherent percept, these two studies do not provide a clear answer to the question if fat perception is a pure taste. Closest to our results, activation of the rostral part of ACC extending to the orbitofrontal cortex by oral fat independently of viscosity has been reported (De Araujo and Rolls, 2004). This region was also activated by sucrose (De Araujo and Rolls, 2004) and in another study by water when thirsty (De Araujo et al., 2003), leading the authors to conclude that this region was sensitive to hedonic properties of diverse stimuli. Here we offer an extended explanation in terms of relevance for the body rather than mere hedonicity.

### Papilla density

A strong correlation between papilla density and sample discrimination by P1 amplitude was found, i.e., the higher the number of papillae, the larger the variability in amplitude of P1 to different stimuli. To our knowledge only one study has indirectly addressed the link between papillae density and brain responses to taste (Eldeghaidy et al., 2011). Taster status assessed by detection threshold for PROP (possibly related to higher papilla density, see Bartoshuk et al., 1994) correlated positively with amplitude of responses in somatosensory, taste, and reward areas as measured with fMRI. EEG offers a direct and precise measurement of cortical responses and therefore our analysis focused on the characteristics of the very first potential P1, which cannot be captured by imaging techniques covering periods of many seconds. At 200 ms after stimulation onset, subjects with higher papilla density had higher discrimination power in terms of amplitude variation of P1 to the different stimuli used. This raises the possibility that high papilla density increases overall sensitivity to different tastes in terms of fine discrimination.

Taken together our findings show that increasing concentration modulates two essential parameters of brain responses (amplitude and latency), which in turn will facilitate stimulus detection as seen in shorter reaction times. Moreover, gERPs confirmed the chronometry of brain activations by taste from primary/gustatory (before ~300–400 ms) to secondary/reward areas (after ~300–400 ms). Fat detection was reflected by a sustained activation of secondary gustatory cortex/reward areas probably reflecting the relevance for the body rather than mere hedonicity of the stimuli. This occurred without explicit recognition of the stimuli by the participants and without altering their intensity judgments for high- and low fat milks, as reflected in identical early cortical responses. Finally, the variability in amplitude of EEG responses to different stimuli was larger for subjects with higher papilla density suggesting an increased discrimination power for these individuals.

One question remains open: by which mechanism is the fat content sensed in the oral cavity? Animal studies have reported behavioral and nerve responses to sweet and umami taste in knock-out mice lacking the T1R3 receptor, which mediates the taste signal (Damak et al., 2003). Recently, another study reported calorie detection in sugars by drosophila mutants lacking sugar receptors Gr5a and Gr64a, after 15 h of food deprivation (Dus et al., 2011). Therefore, though evidence is still needed and underlying mechanisms must be defined, the hypothesis of calorie detection (on the human tongue) independent of taste signaling deserves full attention.

### Conflict of interest statement

The authors declare that the research was conducted in the absence of any commercial or financial relationships that could be construed as a potential conflict of interest.
